# Biomedical Signal and Image Processing for Clinical Decision Support Systems

**DOI:** 10.1155/2013/578320

**Published:** 2013-12-05

**Authors:** Kayvan Najarian, Kevin R. Ward, Shahram Shirani

**Affiliations:** ^1^Department of Computational Medicine and Bioinformatics, Michigan Center for Integrative Research in Critical Care, University of Michigan, Ann Arbor, USA; ^2^Department of Emergency Medicine, Michigan Center for Integrative Research in Critical Care, University of Michigan, Ann Arbor, USA; ^3^Department of Electrical and Computer Engineering, McMaster University, Hamilton, ON, Canada

Clinicians and other health care providers are currently being expected to make an increasing number of consecutive and complex decisions based on a very large amount of complex data collected from a variety of heterogeneous sources, often produced asynchronously. This is complicated by the addition of a growing number of new diagnostic and monitoring devices further highlighting the potential for an ever-growing data stream as well as the challenge of diagnosing and treating multiple patients at one time. It can be argued that there is much hidden knowledge in various clinical data such as images, physiologic signals, and others that simply cannot be rapidly extracted by the human eye. During the last decade, the need for computational methods, in particular signal and image processing algorithms, to analyze these complex data sets and provide health care providers with recommendations and/or predictions has been further highlighted. However, due to the size and complexity of the data produced by monitoring and imaging systems, the need for more effective methods to extract knowledge from these images has not grown with the same rate. This, of course, will impede the development of clinical decision support systems.

This special issue of Computational and Mathematical Methods in Medicine serves as a brief update to the current status of and advances in methods and approaches in biomedical signal and image processing methods used for clinical decision support systems. The computational methods presented in this special issue cover a wide spectrum of algorithmic approaches applied to a wide range of clinical applications.

The paper by X. Li et al. provides a system to identify patients with poststroke mild cognitive impairment by pattern recognition of working memory load-related ERP which may improve our assessment of some of the long-term impacts of stroke. E. Swanly et al. present a methodology to process CT images in order to spot pulmonary TB in a more effective manner. F. Li and F. Porikli give the description of their method that allows tracking of lung tumors in orthogonal X-rays. The paper by N. Saidin et al. presents a method for computer-aided detection of breast density for more accurate detection of breast cancer. This paper also presents a methodology for visualization of other breast anatomical regions on mammogram. H. Jiang et al. provide a hybrid method, based on level-set methods, for extraction of pancreas images from CT scanning with may clinical applications where CT is used for detection of potential damages to pancreas. The study by M. Jiang et al. focuses on parameter optimization for support vector regression in solving the inverse ECG problem, while H.-T. Wu et al. present the results of their study on quantification of the complex fluctuation between R-R intervals series and photoplethysmography amplitude series. Both of these methods may have wide ranging implications for new physiologic diagnostic approaches for patients. The paper presented by Y.-W. Chen et al. focuses on a computer-aided diagnosis and quantification of cirrhotic livers based on morphological analysis and machine learning. I. Cruz-Aceves et al. present their unsupervised cardiac image segmentation that applies multiswarm active contours with a shape prior. Finally, the paper by H. Jiang et al. provides a liver segmentation method, based on snakes model and improved GrowCut algorithm that is applied to abdominal CT images.

As more advanced imaging and monitoring systems are designed, it is expected that the algorithms to process the data produced by these systems need to be evolved accordingly. These novel approaches may not only help extract new knowledge that are not readily possible through current traditional interpretation but also set the stage for providing rapid predictive information assisting health care providers in making better informed decisions. As shown in the papers presented in this special issue, these changes need to address both size and complexity of the produced data. The opportunities are rich as are the challenges.

*Kayvan Najarian* *Kevin R. Ward* *Shahram Shirani*

